# Editorial: The ethics and challenges of studying the genetics of marginalized populations

**DOI:** 10.3389/fgene.2023.1260457

**Published:** 2023-08-22

**Authors:** Tina Lasisi, Katrina G. Claw, Keolu Fox, Arslan A. Zaidi

**Affiliations:** ^1^ Department of Anthropology, University of Michigan, Ann Arbor, MI, United States; ^2^ Division of Biomedical Informatics and Personalized Medicine, Colorado Center for Personalized Medicine, University of Colorado Anschutz Medical Campus, Aurora, CO, United States; ^3^ Department of Anthropology, Global Health Program, University of California, San Diego, La Jolla, CA, United States; ^4^ Genetics, Cell, and Developmental Biology Department, Institute of Health Informatics, University of Minnesota, Saint Paul, MN, United States

**Keywords:** genetics, genomics, ethics, marginalized/vulnerable population, diversity and inclusion

This Research Topic, entitled “*The Ethics and Challenges of Studying the Genetics of Marginalized Populations*,” offers a pointed examination of the ethical considerations embedded within the study of genetics in historically underrepresented groups. Although there is much discourse on the need to include data from historically marginalized groups in genetics research, we believe that true inclusion lies not only in the diversification of samples but also that of researchers. As such, we have made a concerted effort in this Research Topic to invite contributors from diverse backgrounds.

Martschenko and Young open the discussion by challenging precision medicine’s overreliance on broad racial and ethnic classifications. Their provocative critique reminds us that until our collective approach to precision medicine fully engages the complexity and full spectrum of human diversity, it will continue to fall short.

Silva et al.'s case study on the intersection of genomics, mestizaje, and Indigenous identities in Chile further deepens the discourse around the complexities of identity and representation in genetic research. Their cautionary narrative underscores the potential dangers of identity erasure and fetishization of indigeneity, urging the research community to maintain sensitivity towards the social complexities entangled in the field of genomics.

Taking a more environmental lens, Thompson and Crocker critique the common predilection for a genome-focused approach in health studies. They argue that significant health disparities are rooted in environmental factors rather than genetic differences, urging for a comprehensive approach that addresses these social and environmental aspects.

Through their practical work in Zamboanga and the Sulu Archipelago, Rodriguez et al. elucidate the unique ethical challenges associated with conducting genetic research among Indigenous Peoples. Their important work underscores the necessity of respecting the sovereignty of Indigenous Peoples over their genetic information and of developing respectful, equitable research partnerships.

Villanea and Witt address the challenging Research Topic of underrepresented populations in archaic introgression research. Their insightful piece raises vital concerns about potential inequities in research design, highlighting the necessity for equitable research practices and fair benefit sharing.

Jackson et al. provide a necessary critique regarding the underrepresentation of African Americans in genomic studies and present compelling case studies that illustrate the pressing necessity of ensuring broad and fair representation in genetic research.

In their exploration of data sovereignty, Carroll et al. propose the crucial need for Indigenous standards in control and oversight of biomedical data, offering a distinct perspective on the implementation of the CARE Principles for Indigenous Data Governance.

Finally, Young et al. view the challenge of recruiting diverse participants in genomics research through a nuanced lens, highlighting the influence of sociodemographic factors on research participation and emphasizing the importance of various recruitment strategies to ensure diversity.

This set of insightful articles offers a rich palette of perspectives on the myriad ethical challenges faced in genetic studies involving historically marginalized populations. Each piece, while unique in its approach, unites in its mission to shed light on these challenges and deliver potential solutions.

These contributions have critical implications for the wider scientific community, both in terms of advancing our understanding of the genetic architecture of disease risk specifically for non-European (descent) populations, and in highlighting the ethical challenges and proposed solutions for involving and studying marginalized populations. This collective body of work enriches our scientific dialogue, urging us towards more ethical and inclusive research practices.

The contributions illuminate a multifaceted picture of the current status of diverse genetic studies and advocate for a deeper engagement with marginalized populations. The sobering reality of ongoing inequities in the field underscores reliance on past models of research, which are unsatisfactory, and urges a shared vision for the future, where genetic studies are representative, ethical, and equitable.

Importantly, these discussions emphasize the importance of meaningful community involvement, transparent communication, and respect for cultural histories and identities. As we move forward, we must ensure that all populations can participate in, contribute to, and potentially benefit from advances in genetic research. This work underscores the compelling need to continually revisit and reconsider the ethics and practices not only in genetics but in all scientific fields engaging with marginalized populations.

This Research Topic can serve as a foundation to broaden and deepen future research efforts, ultimately enhancing and enlightening our collective understanding of human health and disease.

